# Accurate interval estimation for the risk difference in an incomplete correlated 2 × 2 table: Calf immunity analysis

**DOI:** 10.1371/journal.pone.0272007

**Published:** 2022-07-22

**Authors:** Hezhi Lu, Fengjing Cai, Yuan Li, Xionghui Ou

**Affiliations:** 1 School of Economics and Statistics, Guangzhou University, Guangzhou, 510006, PRC; 2 College of Mathematics and Physics, Wenzhou University, Wenzhou, 325035, PRC; 3 Research Centre for Applied Mathematics, Shenzhen Polytechnic, Shenzhen, 518000, PRC; 4 School of Mathematical Science, South China Normal University, Guangzhou, 510631, PRC; Keio University School of Medicine, JAPAN

## Abstract

Interval estimation with accurate coverage for risk difference (RD) in a correlated 2 × 2 table with structural zero is a fundamental and important problem in biostatistics. The score test-based and Bayesian tail-based confidence intervals (CIs) have good coverage performance among the existing methods. However, as approximation approaches, they have coverage probabilities lower than the nominal confidence level for finite and moderate sample sizes. In this paper, we propose three new CIs for RD based on the fiducial, inferential model (IM) and modified IM (MIM) methods. The IM interval is proven to be valid. Moreover, simulation studies show that the CIs of fiducial and MIM methods can guarantee the preset coverage rate even for small sample sizes. More importantly, in terms of coverage probability and expected length, the MIM interval outperforms other intervals. Finally, a real example illustrates the application of the proposed methods.

## Introduction

An incomplete correlated 2 × 2 table exists in various biological studies, clinical trials and epidemiological studies [1] used this data structure to evaluate penicillin allergy [2,3] studied tuberculosis skin tests in a two-step procedural design. A typical example is given by [4] involving calf immunity data. Calves were first classified according to whether they had a primary pneumonia infection and then reclassified according to whether they developed a secondary infection within a certain time period after the first infection cleared up [5]. Since the subject cannot have secondary infection when it is not infected at the first stage, the cells corresponding to secondary infection without primary infection will not appear. Table 1 lists the observed data and related probabilities.

**Table 1:** Data and probability for an incomplete 2×2 table.

	Secondary infection		
Primary infection	Yes	No	Total
Yes	*X*_11_(*p*_11_)	*X*_12_(*p*_12_)	*X*_1+_(*p*_1+_)
No	−	*X*_22_(*p*_22_)	*X*_22_(*p*_22_)
Total	*X*_11_(*p*_11_)	*X*_12_+*X*_22_(*p*_12_+*p*_22_)	*n*(1)

Suppose there is a sample of *n* subjects, let *X*_11_ be the number of subjects infected in both stages, *X*_12_ be the number of subjects who have a primary infection but do not have a secondary infection, and *X*_22_ be the number of subjects who are not infected in both stages, so that *X*_1+_ = *X*_11_+*X*_12_ and *X*_1+_+*X*_22_ = *n*. Here, *p*_11_, *p*_12_ and *p*_22_ denote the probabilities of the corresponding cells, with *p*_1+_ = *p*_11_+*p*_12_ and *p*_1+_+*p*_22_ = 1. After the first infection clears up, one wants to infer the likelihood of developing a secondary infection due to the effect of the primary infection. One common comparative measure of interest is the risk difference (RD) between the primary infection and the secondary infection, given the primary infection. The RD *δ* is defined by

$$\delta =p_{1+}-p_{11}/p_{1+}.$$

From the frequentist point of view [6], considered confidence intervals (CIs) for the RD based on Wald’s test statistic, the likelihood ratio test and the basic principle of Fieller’s theorem. Although these three approaches behave well in many practical problems, the CIs derived from Wald’s test statistic, the likelihood ratio test and Fieller’s theorem fail to reach the preset confidence level threshold even in moderate sample sizes. As an alternative [2], proposed a score test-based CI and, although the score test statistic is undefined in one scenario, the score CI outperforms all other frequentist intervals in terms of the coverage probability. More literature on risk factors and some potential methods can be found in [6–15].

If a prior distribution is available for the unknown parameter, the Bayesian posterior distribution provides a meaningful summary of the uncertainty about the parameter. To date, the Bayesian approach has been widely used in the interval estimation of the proportions in correlated tables [16] investigated the performance of Bayesian intervals using different priors, and they found that the Jeffreys prior is comparable to the score test-based CI [17] used a Bayesian estimation of the false-negative rate in a clinical trial of a sentinel node biopsy. Moreover [5], used Dirichlet priors to construct the tail-based interval for the RD. Simulation studies showed that the Bayesian CI at the Jeffreys prior has a shorter expected length than the score test-based CI.

The Bayesian choice of priors is a powerful tool for inferring purposes, but different priors can lead to different posterior distributions. To eliminate the influence of the prior distribution on the inference result, the generalized fiducial inference [18,19] is asymptotically correct and works well in applications [20,21]. However, the determination of the fiducial distribution of parameters remains a problem. Unlike the fiducial method, CIs derived from inferential models (IMs) [22–24] can always guarantee nominal coverage for all sample sizes. The main difference between the IM and the fiducial inference is that the IM method always carries out probability calculation in the auxiliary variable space, which can ensure that its inference is strict and correct. Moreover, the IM theory of precise inference needs some improvements. For example [25–29], constructed a randomized IM inference for discrete proportions.

The frequentist methods can be undefined in some cases. Moreover, as two representative CIs, the score and Bayesian intervals cannot guarantee the nominal coverage probability for small to moderate sample sizes. The aim of this paper is to construct three new CIs with accurate coverage for the RD mainly based on the fiducial, IM and randomized IM approaches.

## Existing methods

### Score test-based CI

From Table 1, the observed vector (*X*_11_,*X*_12_) comes from the trinomial distribution model

$$P(X_{11},X_{12}|p_{11},p_{12})=(\begin{array}{c} n\\ X_{11},X_{12} \end{array})p_{11}^{X_{11}}p_{12}^{X_{12}}{\left(1-p_{11}-p_{12}\right)}^{n-X_{11}-X_{12}}.$$ (1)

Let ${\hat{p}}_{1+}=(X_{11}+X_{12})/n$ be the maximum likelihood estimate of *p*_1+_; then, the score test statistic [2] for hypothesis *H*_0_:*δ* = *δ*_0_ is given by

$$T_{S}(X_{11},X_{12},X_{22},\delta_{0})=\frac{X_{1+}({\hat{p}}_{1+}-\delta_{0})-X_{11}}{\left({\hat{p}}_{1+}-\delta_{0})(1-{\hat{p}}_{1+}+\delta_{0}\right)}\sqrt{\frac{1+2\delta_{0}-{\hat{p}}_{1+}^{2}}{n}-\frac{\delta_{0}(1+\delta_{0})}{n{\hat{p}}_{1+}}}.$$

It is well-known that *T*_*S*_(*X*_11_,*X*_12_,*X*_22_,*δ*_0_) is an asymptotically standard normal distribution under *H*_0_. Given the significance level *α*, the two-sided 1−*α* approximate confidence limits (*δ*_*L*_ and *δ*_*U*_) for *δ* can be computed by solving equations

$$T_{S}(X_{11},X_{12},X_{22},\delta_{L})=z_{\alpha /2}\;\mathrm{and}\;T_{S}(X_{11},X_{12},X_{22},\delta_{U})=z_{1-\alpha /2}.$$

The score test is comparable to that of the likelihood ratio test [6], but the likelihood ratio test can be undefined in many scenarios while the score test statistic is undefined when *X*_11_ = *X*_12_ = 0.

### Bayesian tail-based CI

The conditional probability function of (*x*_11_,*x*_12_) given (*p*_11_,*p*_12_) (*p*_11_+*p*_12_<1) is the trinomial distribution with probability function

$$P(x_{11},x_{12}|p_{11},p_{12})=(\begin{array}{c} n\\ x_{11},x_{12} \end{array})p_{11}^{x_{11}}p_{12}^{x_{12}}{\left(1-p_{11}-p_{12}\right)}^{n-x_{11}-x_{12}},$$

where *x*_11_+*x*_12_≤*n*. Recalling Bayes’ rule, if one chooses a prior that is conjugate to the likelihood, then the posterior will have the same form as the conjugate prior distribution. Clearly, the Dirichlet *D*_3_(*k*_1_,*k*_2_,*k*_3_) prior for the parameter (*p*_11_,*p*_12_) leads to the posterior being a Dirichlet-type distribution *D*_3_(*x*_11_+*k*_1_,*x*_12_+*k*_2_,*n*−*x*_11_−*x*_12_+*k*_3_) with the posterior density function

$$f(p_{11},p_{12}|x_{11},x_{12})\propto p_{11}^{x_{11}+k_{1}-1}p_{12}^{x_{12}+k_{2}-1}{\left(1-p_{11}-p_{12}\right)}^{n-x_{11}-x_{12}+k_{3}-1}.$$

Shi, Sun and Bai [5] studied the symmetric Dirichlet prior *k*_*i*_ = *k*, *i* = 1,2,3 for the RD. They recommended the Jeffreys prior *D*_3_(1/2,1/2,1/2). Moreover, the posterior distribution function of *δ* given (*x*_11_,*x*_12_) has the following expression

$$F(\delta |x_{11},x_{12})=\{\begin{array}{c} c(x,k)J_{x_{11}+x_{12}+2k,n-x_{11}-x_{12}+k,x_{12}+k,x_{11}+k}(\delta ),\;-1<\delta <0,\\ 1-c(x,k)J_{n-x_{11}-x_{12}+k,x_{11}+x_{12}+2k,x_{11}+k,x_{12}+k}(\delta ),\;0\leq \delta <1, \end{array}$$ (2)

where $c(x,k)=\Gamma (n+3k)/[\Gamma (x_{11}+k)\Gamma (x_{12}+k)\Gamma (n-x_{11}-x_{12}+k)]$ and $J_{m_{1},m_{2},m_{3},m_{4}}(\delta )$ for *m*_*i*_>0, *i* = 1,2,3,4 is defined by

$$J_{m_{1},m_{2},m_{3},m_{4}}(\delta )={\left(1+\delta \right)}^{m_{1}}\int_{0}^{1}t^{m_{1}-1}{\left[1-(1+\delta )t\right]}^{m_{2}-1}g_{m_{3},m_{4}}((1+\delta )(1-t))dt,$$ (3)

where $g_{m_{3},m_{4}}(y)=\int_{0}^{y}u^{m_{3}-1}{\left(1-u\right)}^{m_{4}-1}du$. For the tail-based CI with a significance level *α*, the lower and upper limits (*L*,*U*) are obtained by minimizing the following function

$$G(L,U)=|F(U|x_{11},x_{12})-(1-\alpha /2)|+|F(L|x_{11},x_{12})-\alpha /2|.$$ (4)

Note that mathematical calculations of the integral formulae (3) and function (4) require some specific algorithms, such as the Nelder–Mead algorithm.

## New confidence intervals

The small sample properties for approximate score and Bayesian methods may not be calibrated for meaningful probabilistic inference. These two CIs have lower coverage probability in some cases. To date, fiducial and IM-based methods have been used in other inference problems where classic approaches cannot lead to valid inference. Therefore, we consider fiducial and IM solutions for the RD.

### Fiducial CI

Fiducial inference is based entirely on the fiducial distribution of the parameters. We first review the fiducial framework [18]. Suppose that *X* is a random variable indexed by a parameter *θ* which is generated using the structural equation given by

X=a(θ,U),

where *a* is a measurable function and *U* is an auxiliary variable with a known distribution. Note that the distribution of *U* is free of *θ*.

Let *x* and *u* be realization of *X* and *U*, respectively. The information provided by *x* and *u* about *θ* is encapsulated in the set

Θ(x,u)={θ|x=a(θ,u)}.

Clearly, *x* = *a*(*θ*,*u*) is equivalent to Θ(*x*,*u*)≠∅. The fiducial argument involves replacing *u* with *U*’ to obtain the random set Θ(*x*,*U*’), where *U*’ is an independent copy of *U*. Since the fiducial distribution of *θ* is given by

$$\theta =\Theta (x,U^{'})|\Theta (x,U^{'})\neq \emptyset .$$

Then, we can easily derive a fiducial CI for *θ*.

From Table 1, if the condition is based on the number (*x*_11_+*x*_12_), such that all marginal totals are fixed, the probability of observing *x*_11_ follows a binomial distribution. By derivation, letting *T* = *X*_11_+*X*_12_ and *X* = *X*_11_, the probability function of (*x*_11_,*x*_12_) in (1) can be simplified as the product of two independent binomial distributions as follows:

$$P\{x_{11},x_{12};p_{11},p_{12}\}=P\{T=t;\theta \}\cdot P\{X=x|T=t;\varphi \},$$ (5)

where *θ* = *p*_11_+*p*_12_ and *φ* = *p*_11_/(*p*_11_+*p*_12_).

Naturally, we have *T*~*Bin*(*n*,*θ*) and *X*|*T* = *t*~*Bin*(*t*,*φ*). Moreover, if we define the value of the random variable to be 1 if a trial results in success, and 0 otherwise, then the structural equation can be given by

$$T=\sum_{i=1}^{n}{Ι}_{\left[0,\theta \right]}(U_{i})\;\mathrm{and}\;X=\sum_{j=1}^{t}{Ι}_{\left[0,\varphi \right]}(V_{j})$$

where I_*A*_(⋅) is the indicator function and *U*_*i*_, *i* = 1,…,*n*, and *V*_*j*_, *j* = 1,…,*t*, are independent uniform(0,1) random variables.

Due to the discrete nature, event $A_{k_{1}}=\{T=k_{1}\}$ is equivalent to event $B_{k_{1}}=\{\sum_{i=1}^{k_{1}}U_{i}\leq \theta <\sum_{i=1}^{k_{1}+1}U_{i}\}$. Similarly, event $A_{k_{2}}=\{X=k_{2}\}$ is equivalent to event $B_{k_{2}}=\{\sum_{j=1}^{k_{2}}V_{j}\leq \varphi <\sum_{j=1}^{k_{2}+1}V_{j}\}$. Given *T* = *t* and *X* = *x*, we have

$$\sum_{i=1}^{t}U_{i}\leq \theta <\sum_{i=1}^{t+1}U_{i}\;\mathrm{and}\;\sum_{j=1}^{x}V_{j}\leq \varphi <\sum_{j=1}^{x+1}V_{j}.$$

Moreover, the fiducial quantity for the RD δ=θ−φ is given by

$$\sum_{i=1}^{t}U_{i}-\sum_{j=1}^{x+1}V_{j}\leq \delta \leq \sum_{i=1}^{t+1}U_{i}-\sum_{j=1}^{x}V_{j}.$$ (6)

Since we can easily obtain a Monte Carlo approximation for the distribution of the two endpoints, constructing a fiducial CI for *δ* is not problematic.

***Remark 1*.** Compared to Bayesian inference, the fiducial idea may be more attractive because no prior distributions are needed. Moreover, the fiducial idea has been shown to be asymptotically correct for a single discrete population. However, there is little research on risk factors between two independent binomial distributions. As an extension, our research can fill this gap and is easily applied to other multiparameter inference problems. Furthermore, our simulation studies will show that the fiducial method can guarantee nominal coverage even for small sample sizes.

### IM-based CI

Martin and Liu [22] proposed an IM framework for valid probabilistic inference. The IM starts with an association between data, parameters and auxiliary variables. Using the optimal predictive random set to predict the auxiliary variables, the IM produces a postdata probabilistic measure function of uncertainty about the unknown parameter. To summarize, the IM has the following three steps:

*A*-step: From an appropriate mapping *a*: *x* = *a*(*θ*,*U*), the IM associates the parameter *θ* with (*x*,*u*) for each possible pair to obtain a collection of sets Θ_*x*_(*u*) of candidate values.

*P*-step: Given data *x* , suppose θ* is the true value of *θ*, there exists a u*, such that *x* = *a*(θ*,u*). Moreover, the true value u* is predicted with a valid predictive random set *S*(*u*). The validity condition ensures that *S*(*u*) will hit u* with large probability.

*C*-step: The *A*-step and *P*-step are combined to obtain a final random set of *θ*, that is, Θ_*x*_(*S*(*u*)) = ∪_*u*∈*S*(*u*)_Θ_*x*_(*u*). Then, for any assertion *A* about the parameter of interest *θ*, the probabilities that *bel*_*x*_(*A*) = *P*{Θ_*x*_(*S*(*u*))⊂*A*} and *pl*_*x*_(*A*) = *P*{Θ_*x*_(*S*(*u*))⊄*A*^*c*^} are computed as two measure functions of the available evidence in *x* supporting *A*.

The belief function *bel*_*x*_(*A*) and the plausibility function *pl*_*x*_(*A*) are known as the minimum and maximum probabilities that support the truth of assertion *A*. It is more convenient to report the plausibility function, which can easily be used to create frequentist procedures. To test the assertion *A* = {*δ*:*δ* = *δ*_0_}, we reject *H*_0_:*δ* = *δ*_0_ if *pl*_*x*_(*A*)≤*α* for a significance level *α*, and this plausibility function yields a two-sided 1−*α* IM CI {*δ*: *pl*_*x*_(*A*)>*α*}.

***Theorem 1*** *[22].* Suppose *X*~*P*_*X*|*θ*_ and let *A* be an assertion of interest, the IM with the plausibility function *pl*_*X*_(*A*) is valid for assertion *A* if, for each *α*∈(0,1),

$$\underset{\theta \in A}{\sup}P_{X|\theta }\{pl_{X}(A)\leq \alpha \}\leq \alpha .$$

The resulting IM CI can guarantee the nominal coverage probability when the plausibility function *pl*_*X*_(*A*) is said to be valid. Moreover, if "≤*α*" can be replaced by "= *α*", then the IM CI controls the coverage probability exactly at the confidence level 1−*α*.

Here, we construct a new IM CI for the RD. Let *F*_*n*,*θ*_(⋅) denote the distribution function of *T*~*Bin*(*n*,*θ*). Martin and Liu [22] gave an association linking *t*, *θ*, and an auxiliary variable *u*~*P*_*u*_ as follows:

$$t=\min\{k:u<F_{n,\theta }(k)\},\;u∼Unif(0,1).$$ (7)

Moreover, the association model (7) can be simplified as

$$F_{n,\theta }(t-1)\leq u<F_{n,\theta }(t),\;u∼Unif(0,1).$$ (8)

Similarly, the association for *x*, given *φ*, may be written as

$$F_{t,\varphi }(x-1)\leq v<F_{t,\varphi }(x),\;v∼Unif(0,1).$$ (9)

To derive an initial IM’s association for *δ* = *θ*−*φ*, we will take advantage of the well-known relationship between the binomial and beta distribution functions, that is, $F_{n,\theta }(t)=1-G_{t+1,n-t}(\theta )$, where *G*_*a*,*b*_(⋅) is the beta (a, b) distribution function. Furthermore, we can rewrite joint associations (8) and (9) as follows:

$$G_{t,n-t+1}^{-1}(u)\leq \theta <G_{t+1,n-t}^{-1}(u)\;\mathrm{and}\;G_{x,t-x+1}^{-1}(v)\leq \varphi <G_{x+1,t-x}^{-1}(v),$$

where *u* and *v* are i.i.d. uniform(0,1) random variables. Hence, the *A*-step of the IM for *δ* is

$$G_{t,n-t+1}^{-1}(u)-G_{x+1,t-x}^{-1}(v)\leq \delta <G_{t+1,n-t}^{-1}(u)-G_{x,t-x+1}^{-1}(v).$$ (10)

***A*-step**: Let *H*_*t*−1,*x*_(⋅) and *H*_*t*,*x*−1_(⋅) be the distribution functions of the two endpoints in (10). The association step of IM for the RD is

$$\Theta_{t,x}(w)=\{\delta :H_{t-1,x}^{-1}(w)\leq \delta <H_{t,x-1}^{-1}(w)\},\;w∼Unif(0,1).$$

***P*-step**: We are interested in two-sided CIs. Following [22], for a singleton assertion *A* = {*δ*}, the default predictive random set for the auxiliary variable *w*, S={w:|w−0.5|≤|W−0.5|},

W∼Unif(0,1).

***C*-step**: Combine Θ_*t*,*x*_(*w*) and *S* to obtain a random set $\Theta_{t,x}(S)=\cup_{w\in S}\Theta_{t,x}(w)$ for *δ*. Since $\delta \notin \Theta_{t,x}(S)\Leftrightarrow H_{t,x-1}(\delta )>\sup S$ or *H*_*t*−1,*x*_(*δ*)<inf *S*,

we find that the corresponding plausibility function for a singleton assertion *A* = {*δ*} is

$$\begin{array}{l} pl_{t,x}(A)=P_{S}\{\delta \in \Theta_{t,x}(S)|\Theta_{t,x}(S)\neq \emptyset \}\\ \;=1-P_{W}\{0.5+|W-0.5|<H_{t,x-1}(\delta )\cup 0.5-|W-0.5|>H_{t-1,x}(\delta )\}\\ \;=1-P_{W}\{|2W-1|<2H_{t,x-1}(\delta )-1\}-P_{W}\{|2W-1|<1-2H_{t-1,x}(\delta )\}\\ \;=1-{\left\{2H_{t,x-1}(\delta )-1\right\}}^{+}-{\left\{1-2H_{t-1,x}(\delta )\right\}}^{+}, \end{array}$$

where the ‘‘+” superscript denotes the positive part.

***Theorem 2***. According to Theorem 1, the plausibility function *pl*_*t*,*x*_(*A*) of our IM method is valid for assertion *A* if, for each *α*∈(0,1),

$$\underset{\delta \in A}{\sup}P_{T,X|\delta }\{pl_{T,X}(A)\leq \alpha \}\leq \alpha .$$

***Proof*.** Given any *α*∈(0,1), we have

$$\begin{array}{l} \;P_{T,X|\delta }\{pl_{T,X}(A)\leq \alpha \}\\ =P_{T,X|\delta }\{1-{\left\{2H_{t,x-1}(\delta )-1\right\}}^{+}-{\left\{1-2H_{t-1,x}(\delta )\right\}}^{+}\leq \alpha \}\\ =P_{T,X|\delta }\{1-(2H_{t,x-1}(\delta )-1)\leq \alpha ,H_{t,x-1}(\delta )\geq 1/2\}+P_{T,X|\delta }\{2H_{t-1,x}(\delta )\leq \alpha ,H_{t-1,x}(\delta )\leq 1/2\}\\ =P_{T,X|\delta }\{H_{t,x-1}(\delta )\geq 1-\alpha /2,H_{t,x-1}(\delta )\geq 1/2\}+P_{T,X|\delta }\{H_{t-1,x}(\delta )\leq \alpha /2,H_{t-1,x}(\delta )\leq 1/2\}. \end{array}$$

Then, $\underset{\delta \in A}{\sup}P_{T,X|\delta }\{pl_{T,X}(A)\leq \alpha \}\leq \alpha .$ Hence, the proof is complete.

For any *α*∈(0,1), if *pl*_*t*,*x*_(*A*)≤*α*, then the assertion *A* is wrong. Moreover, this plausibility function yields an IM 1−*α* CI {*δ*: *pl*_*t*,*x*_(*A*)>*α*}, such that *δ*∈[*δ*_*L*_,*δ*_*U*_], where *δ*_*L*_ and *δ*_*U*_ satisfy

$$H_{t-1,x}(\delta_{L})=\alpha /2,\;H_{t,x-1}(\delta_{U})=1-\alpha /2.$$

***Remark 2*.** The IM CI has the same form as the generalized fiducial CI. However, these two intervals are obtained under different theoretical derivations, fiducial approaches and IM theories, respectively. While the general fiducial distributions in (6) may not be calibrated for meaningful probabilistic inference, IM provides meaningful probabilistic summaries of the information in data concerning the quantity of interest. Moreover, the IM CI derived from a valid plausibility function of IM can guarantee the nominal coverage probability for all sample sizes.

### Modified IM CI

In general, the association model (10) of IM is an interval, which will result in a conservative CI. Some adjustments are needed to handle this discreteness. Inspired by the randomized IM idea [25], we consider a modified IM (MIM) approach to modify in Eq (10) to an accurate equation so that we can improve the accuracy of the candidate value of *δ*.

***Theorem 3*** *[25].* Suppose *Y*~*Bin*(*m*,*ϕ*), let *ω* be uniformly distributed in (0,1), and *Y* and *ω* are independent. Then $\omega F_{m,\theta }(y-1)+(1-\omega )F_{m,\theta }(y)∼Unif(0,1).$

For association (8), since the auxiliary variable *u* is in the interval $\left[F_{n,\theta }(t-1),F_{n,\theta }(t)\right)$, there exists a weight *ω*_1_ such that

$$u=\omega_{1}F_{n,\theta }(t-1)+(1-\omega_{1})F_{n,\theta }(t),$$ (11)

where *ω*_1_ follows a uniform(0,1) distribution and is independent of *t*. Note that $\omega_{1}F_{n,\theta }(t-1)+(1-\omega_{1})F_{n,\theta }(t)$ is a strictly decreasing function of *θ*, for every *u*,*ω*_1_∈(0,1), we can obtain a unique solution $\theta =G_{t}^{'}(u,\omega_{1})$ from Eq (11). Moreover, let its distribution function be *G*_*t*_(⋅); then, the association model for *θ* can be rewritten as follows:

$$\theta =G_{t}^{-1}(u^{'}),\;u^{'}∼Unif(0,1).$$ (12)

Similarly, for inequality (9), if *ω*_2_~*Unif*(0,1) and is independent of *x*, then we obtain

$$v=\omega_{2}F_{t,\varphi }(x-1)+(1-\omega_{2})F_{t,\varphi }(x).$$ (13)

Given *x*, for every *v*,*ω*_2_∈(0,1), Eq (12) gives a unique solution $\varphi =H_{x}^{'}(v,\omega_{2})$. Let its distribution function be *H*_*x*_(⋅); then, the association model for *φ* is

$$\varphi =H_{x}^{-1}(v^{'}),\;v^{'}∼Unif(0,1).$$ (14)

***A’*-step**: Based on (12) and (14), our new association step of the MIM for the RD *δ* = *θ*−*φ* is

$$\Theta_{t,x}^{'}(w)=\{\delta :\delta =G_{t}^{-1}(u^{'})-H_{x}^{-1}(v^{'})=K_{t,x}^{-1}(w)\},\;w∼Unif(0,1),$$

where *K*_*t*,*x*_(⋅) is the distribution function of $G_{t}^{-1}(u^{'})-H_{x}^{-1}(v^{'})$.

***P’*-step**: For a singleton assertion *A*, the default predictive random set for the auxiliary variable *w* is

S={w:|w−0.5|≤|W−0.5|},W∼Unif(0,1).

***C’*-step**: To obtain a final random set for *δ*, $\Theta_{t,x}^{'}(w)$ and *S* are combined as

$$\Theta_{t,x}^{'}(S)=\cup_{w\in S}\Theta_{t,x}^{'}(w)=[K_{t,x}^{-1}(0.5-|W-0.5|),K_{t,x}^{-1}(0.5+|W-0.5|)],$$

where *W*~*Unif*(0,1). Then, we can compute the plausibility function of *A*, that is, $\begin{array}{l} pl_{t,x}^{'}(A)=\mathrm{Pr}\{\Theta_{t,x}^{'}(S)\cap A\neq \emptyset |\Theta_{t,x}^{'}(S)\neq \emptyset \}\\ \;=\mathrm{Pr}\{W\leq 0.5-|K_{t,x}(\delta )-0.5|\cup W\geq 0.5+|K_{t,x}(\delta )-0.5|\}\\ \;=1-|2K_{t,x}(\delta )-1| \end{array}$.

***Theorem 4*.** Let *S*~*P*_*S*_ be a valid predictive random set for *W*~*Unif*(0,1), that is, P_*S*_(*w*∈*S*)≥_*st*_*Unif*(0,1), where "≥_*st*_" means “stochastically no smaller than”. If *K*_*t*,*x*_(*δ*)~*Unif*(0,1) for (*t*,*x*)~*P*_(*t*,*x*)|*δ*_ for all *δ*, then the MIM method is valid.

***Proof*.** Given any *α*∈(0,1), since *K*_*t*,*x*_(*δ*)~*Unif*(0,1) for (*t*,*x*)~*P*_(*t*,*x*)|*δ*_ for all *δ*, and

$$pl_{t,x}^{'}(A)=P_{S}\{\Theta_{t,x}^{'}(S)\cap A\neq \emptyset \}=P_{S}\{K_{t,x}(\delta )\in S\}=P_{S}\{w\in S\}.$$

Moreover, the predictive random set *S*~*P*_*S*_ is valid, that is, P_*S*_(*w*∈*S*)≥_*st*_*Unif*(0,1).

Hence, $\underset{\delta \in A}{\sup}\;P_{(t,x)|\delta }\{pl_{t,x}^{'}(A)\leq \alpha \}=\underset{\delta \in A}{\sup}\;P_{(t,x)|\delta }\{P_{S}\{w\in S\}\leq \alpha \}$

$$\leq \underset{\delta \in A}{\sup}\;P_{(t,x)|\delta }\{Unif(0,1)\leq \alpha \}=\alpha .$$

The MIM inference is valid, by Theorem 1. Hence, the proof is complete.

The plausibility function of our MIM method yields a new CI $\left[\delta_{L},\delta_{U}]=\{\delta :pl_{t,x}^{'}(A)>\alpha \right\}$, where *δ*_*L*_ and *δ*_*U*_ satisfy *K*_*t*,*x*_(*δ*_*L*_) = *α*/2 and *K*_*t*,*x*_(*δ*_*U*_) = 1−*α*/2.

***Remark 3*.** Similar to the classical approaches, the Monte Carlo method is also an approximate solution. The main difference between our MIM approach and other approximations is that the accuracy of MIM depends on the repetition times *N*, but accuracies of other approximations depend on the sample size *n*. We recommend *N =* 1,000,000 in practical applications to assure that there is a greater than 95% probability of the absolute error being less than 0.001.

## Simulation results

The fiducial and IM CIs have the same form. The score, Bayesian, fiducial and MIM approaches are approximations. We conduct some Monte Carlo simulations to assess the performance of fiducial and MIM intervals, and compare them to the score and Bayesian intervals. Since it is often difficult to obtain explicit expressions of the fiducial and MIM CIs, we suggest approximating these two CIs using the following Monte Carlo algorithms. R codes are available in the S1 Appendix.

Table 2 lists four 95% CIs for various combinations including some special cases of zero cells. We see that the Bayesian, fiducial and MIM intervals are well defined for all cases. However, when *X*_11_ = *X*_12_ = 0, the score interval does not exist and the fiducial and MIM CIs have shorter widths than the score and Bayesian CIs. Note that the expected lengths of the score, Bayesian and MIM intervals are almost the same when the sample size increases.

**Algorithm 1: Fiducial CI (*δ*_*α*/2_** , *δ*_1−*α*/2_ **)**

Step 1. For the given sample (*x*_11_,*x*_12_) from the incomplete correlated 2 × 2 table,

    *t* = *x*_11_+*x*_12_ and *x* = *x*_11_ are calculated;

Step 2. Then, *u*_1_,*u*_2_,…,*u*_*t*+1_ and *v*_1_,*v*_2_,…,*v*_*x*+1_ are generated from a uniform(0,1) distribution,

    and *δ* is calculated using $\sum_{i=1}^{t}u_{i}-\sum_{j=1}^{x+1}v_{j}\leq \delta \leq \sum_{i=1}^{t+1}u_{i}-\sum_{j=1}^{x}v_{j}$;

Step 3. Step 2 is repeated *N* times (1,000,000 for example) to obtain *N* realizations of *δ*;

Step 4. The *α*/2 quantile of $\sum_{i=1}^{t}U_{i}-\sum_{j=1}^{x+1}V_{j}$ and the 1−*α*/2 quantile of

    $\sum_{i=1}^{t+1}U_{i}-\sum_{j=1}^{x}V_{j}$ are calculated to approximate *δ*_*α*/2_ and *δ*_1−*α*/2_, respectively.

**Algorithm 2: MIM CI (***δ*_*α*/2_, *δ*_1−*α*/2_**)**

Step 1. For the given sample (*x*_11_,*x*_12_), *t* = *x*_11_+*x*_12_ and *x* = *x*_11_ are calculated;

Step 2. Then *ω*_1_, *ω*_2_, *u* and *v* are randomly sampled from the uniform(0, 1) distribution, and

    equations $u=\omega_{1}F_{n,\theta }(t-1)+(1-\omega_{1})F_{n,\theta }(t)$ and $v=\omega_{2}F_{t,\varphi }(x-1)+(1-\omega_{2})F_{t,\varphi }(x)$ are solved to obtain the unique solution (*θ*,*φ*). Then, *δ* = *θ*−*φ* is calculated;

Step 3. Step 2 is repeated *N* times (1,000,000 for example) to obtain *N* realizations of *δ*;

Step 4. The *α*/2 and 1−*α*/2 quantiles of *δ* are calculated to approximate *δ*_*L*_ and *δ*_*U*_,

    respectively.

**Table 2:** The four 95% confidence intervals and widths for the selected combinations of (*n*,*X*_11_,*X*_12_).

*n*	*X*_11_	*X*_12_	Score	Bayesian	Fiducial	MIM
5	0	0	- -	(-0.990, 0.830) 1.820	(-1.000, 0.519) 1.519	(-1.000, 0.371) 1.371
5	5	0	(-0.301, 0.329) 0.630	(-0.415, 0.457) 0.872	(-0.522, 0.522) 1.044	(-0.406, 0.406) 0.812
5	2	0	(-0.847, 0.352) 1.199	(-0.805, 0.217) 1.022	(-0.946, 0.460) 1.406	(-0.897, 0.294) 1.191
5	0	2	(-0.300, 0.754) 1.054	(-0.290, 0.744) 1.034	(-0.607, 0.854) 1.461	(-0.457, 0.774) 1.231
30	1	20	(0.432, 0.804) 0.372	(0.425, 0.800) 0.375	(0.371, 0.825) 0.454	(0.411, 0.803) 0.392
50	20	5	(-0.477, -0.056) 0.421	(-0.477, -0.068) 0.409	(-0.507, -0.040) 0.467	(-0.486, -0.068) 0.418
50	10	10	(-0.338, 0.155) 0.493	(-0.329, 0.163) 0.492	(-0.374, 0.182) 0.556	(-0.347, 0.153) 0.500

For comparison, simulation studies are conducted to examine the performances of the score, Bayesian, fiducial and MIM CIs under different sample sizes. The parameters of the comparison are mainly the coverage probability and expected length. Refer to [2,5] for the parameter settings. We consider *p*_1+_ = 0.3, 0.5 and 0.8; *δ* = −0.3 (0.1) 0.3; and *n* = 20, 50, 100 and 300. In each simulation, we first resample the observed value (*X*_11_,*X*_12_) 10,000 times from the trinomial distribution, calculate the four different CIs accordingly, and compute the corresponding frequencies that cover *δ*. We regard the coverage frequency as the coverage probability. According to the central limit theorem, the coverage frequency of the nominal 95% confidence level tends to fall in the interval (0.9457, 0.9543) for 10,000 experimental repetitions. Moreover, the expected length is

$$\begin{array}{l} Expected\;length=E_{n,p_{11},p_{12}}(length(CI))\\ \;=\sum_{\left(X_{11},X_{12}\right)}(\delta_{U}-\delta_{L})\cdot (\begin{array}{c} n\\ X_{11},X_{12} \end{array})p_{11}^{X_{11}}p_{12}^{X_{12}}{\left(1-p_{11}-p_{12}\right)}^{n-X_{11}-X_{12}} \end{array}$$

where *δ*_*U*_ and *δ*_*L*_ are the upper and lower limits of the interval, respectively.

We report the simulation results in Tables 3 and 4. Cases in which the coverage probability is less than 0.9457 appear in bold underlined. Clearly, the score and Bayesian CIs cannot guarantee the nominal coverage probability for small to moderate samples, except for the fiducial and MIM CIs. Moreover, the score and MIM intervals have similar coverage in most cases, but the expected lengths of score CIs are longer than those of MIM CIs. For example, when *n* = 20, *p*_1+_ = 0.3 and *δ* = 0.2, the coverage rates of the score and MIM CIs are 0.9647 and 0.9679, respectively. In this case, the expected lengths of the score CI (0.93) are significantly longer than those of the MIM CI (0.70). Furthermore, it seems that the score CI has a more accurate coverage probability than the MIM CI in some cases such as *n* = 50, *p*_1+_ = 0.3 and *δ* = 0.1, but our MIM CI also has a shorter length than the score CI. Note that our MIM method uses a shorter interval to obtain higher coverage, which shows that the MIM CI outperforms the score CI.

**Table 3:** The coverage probability (CP) and the expected length (EL) of various 95% CIs.

			Score		Bayesian		Fiducial		MIM	
*n*	*p*_1+_	*δ*	CP	EL	CP	EL	CP	EL	CP	EL
20	0.3	-0.3	96.06	0.78	96.06	0.74	98.93	0.90	97.02	0.78
		-0.2	96.02	0.79	**93.29**	0.75	98.96	0.91	95.32	0.79
		-0.1	**94.31**	0.80	**94.31**	0.74	98.71	0.91	96.34	0.79
		0.0	95.02	0.82	**94.03**	0.72	98.88	0.89	96.44	0.77
		0.1	96.60	0.85	96.15	0.69	98.62	0.87	96.50	0.74
		0.2	96.47	0.93	95.81	0.64	98.78	0.83	96.79	0.70
	0.5	-0.3	95.97	0.68	95.49	0.61	98.53	0.75	97.28	0.64
		-0.2	95.77	0.68	**93.76**	0.65	98.93	0.78	95.24	0.67
		-0.1	95.13	0.68	95.13	0.67	97.75	0.80	95.94	0.69
		0.0	**93.56**	0.68	**93.41**	0.68	98.26	0.81	95.62	0.69
		0.1	94.84	0.68	**93.76**	0.67	98.39	0.80	95.76	0.70
		0.2	95.20	0.66	**93.36**	0.65	98.40	0.78	94.86	0.69
		0.3	95.34	0.65	94.58	0.61	98.34	0.75	95.26	0.67
	0.8	-0.1	96.15	0.60	95.70	0.45	99.23	0.56	96.44	0.47
		0.0	95.57	0.55	**94.45**	0.50	98.07	0.61	95.71	0.52
		0.1	95.03	0.55	94.60	0.53	98.06	0.64	95.34	0.55
		0.2	94.92	0.56	94.79	0.55	98.03	0.65	95.54	0.56
		0.3	95.04	0.56	**93.85**	0.55	97.68	0.66	95.39	0.57
50	0.3	-0.3	94.84	0.52	95.07	0.52	97.70	0.59	95.73	0.53
		-0.2	95.37	0.53	**94.49**	0.52	97.42	0.60	95.52	0.53
		-0.1	95.15	0.52	**94.46**	0.52	97.56	0.59	95.28	0.53
		0.0	94.61	0.50	94.66	0.50	97.80	0.57	95.68	0.51
		0.1	95.11	0.49	95.05	0.46	97.99	0.54	95.84	0.47
		0.2	95.36	0.54	95.00	0.40	98.42	0.48	96.08	0.42
	0.5	-0.3	95.14	0.42	94.85	0.40	97.17	0.46	94.97	0.41
		-0.2	95.26	0.44	**94.43**	0.43	97.42	0.49	95.36	0.44
		-0.1	94.79	0.46	94.89	0.45	96.97	0.51	95.34	0.46
		0.0	95.01	0.46	95.11	0.46	97.11	0.51	95.64	0.46
		0.1	95.18	0.45	95.38	0.45	97.02	0.51	95.48	0.46
		0.2	94.79	0.44	94.73	0.43	97.17	0.49	95.05	0.44
		0.3	94.60	0.41	94.75	0.40	97.23	0.46	95.21	0.41
	0.8	-0.1	95.31	0.31	**94.48**	0.29	97.74	0.33	95.27	0.29
		0.0	95.62	0.34	95.02	0.33	97.34	0.37	95.25	0.33
		0.1	95.48	0.36	94.91	0.35	97.38	0.39	95.11	0.36
		0.2	95.05	0.37	94.85	0.36	97.30	0.41	95.07	0.37
		0.3	94.99	0.37	94.80	0.37	97.31	0.41	95.30	0.37

**Table 4:** The coverage probability (CP) and the expected length (EL) of various 95% CIs.

			Score		Bayesian		Fiducial		MIM	
*n*	*p*_1+_	*δ*	CP	EL	CP	EL	CP	EL	CP	EL
100	0.3	-0.3	94.90	0.38	94.62	0.38	96.99	0.42	94.93	0.38
		-0.2	95.17	0.39	94.88	0.39	96.96	0.43	94.94	0.39
		-0.1	95.05	0.38	94.71	0.38	97.09	0.42	95.11	0.38
		0.0	94.81	0.36	94.92	0.36	96.68	0.40	95.25	0.36
		0.1	95.41	0.33	94.65	0.33	97.09	0.37	95.30	0.33
		0.2	95.16	0.28	94.97	0.28	98.15	0.32	94.88	0.28
	0.5	-0.3	95.04	0.29	94.80	0.29	96.95	0.32	95.07	0.29
		-0.2	95.16	0.31	94.98	0.31	96.72	0.34	95.20	0.31
		-0.1	94.89	0.33	94.94	0.33	97.06	0.36	95.01	0.33
		0.0	95.04	0.33	95.05	0.33	96.44	0.36	95.10	0.33
		0.1	95.08	0.33	94.75	0.33	96.62	0.36	95.10	0.33
		0.2	94.89	0.31	94.94	0.31	96.44	0.34	94.76	0.31
		0.3	94.90	0.29	94.90	0.29	96.92	0.32	94.80	0.29
	0.8	-0.1	95.11	0.20	94.88	0.20	97.11	0.22	94.70	0.20
		0.0	95.02	0.23	94.74	0.23	96.57	0.25	95.04	0.23
		0.1	95.28	0.25	94.97	0.25	96.55	0.27	94.78	0.25
		0.2	95.15	0.26	94.98	0.26	96.74	0.28	95.10	0.26
		0.3	95.00	0.26	95.00	0.26	96.43	0.29	95.00	0.26
300	0.3	-0.3	94.81	0.23	94.82	0.23	96.32	0.24	94.91	0.23
		-0.2	95.13	0.23	94.93	0.23	96.34	0.24	95.03	0.23
		-0.1	95.29	0.23	95.01	0.23	96.25	0.24	95.19	0.23
		0.0	95.08	0.21	95.38	0.21	96.59	0.23	95.18	0.21
		0.1	94.99	0.19	94.79	0.19	95.94	0.21	94.99	0.19
		0.2	95.07	0.16	95.26	0.16	96.84	0.17	95.17	0.16
	0.5	-0.3	94.99	0.17	94.99	0.17	95.87	0.18	94.89	0.17
		-0.2	94.83	0.18	94.92	0.18	95.79	0.19	94.83	0.18
		-0.1	94.70	0.19	94.79	0.19	95.96	0.20	94.80	0.19
		0.0	94.86	0.19	94.96	0.19	95.76	0.20	94.86	0.19
		0.1	94.88	0.19	94.88	0.19	95.65	0.20	94.78	0.19
		0.2	94.89	0.18	95.01	0.18	95.98	0.19	94.99	0.18
		0.3	94.96	0.17	94.97	0.17	96.14	0.18	94.97	0.17
	0.8	-0.1	94.88	0.12	94.94	0.12	96.12	0.13	94.83	0.12
		0.0	95.16	0.14	95.12	0.14	96.20	0.14	95.07	0.14
		0.1	95.02	0.15	95.26	0.15	96.08	0.15	95.22	0.15
		0.2	94.98	0.15	95.03	0.15	95.87	0.16	95.12	0.15
		0.3	94.93	0.16	95.17	0.16	96.19	0.16	95.21	0.16

From Table 4, when the sample size increases, the coverage probabilities of the score, Bayesian and modified IM CIs all fall in the interval (0.9457, 0.9543). Although the fiducial CI has a slightly larger coverage rate than other intervals, the expected lengths of the four CIs are almost the same. In this sense, the fiducial method is not inferior to existing methods. Moreover, according to the central limit theorem, the large sample properties indicate that the theoretical results of the score, Bayesian, fiducial and modified IM methods will tend to be consistent. In summary, the fiducial and MIM methods can improve the poor coverage probabilities of the score and Bayesian approaches for small to moderate sample sizes. For larger samples, the CIs of score, Bayesian and MIM are the same, and the fiducial interval is not inferior to the other three intervals. Compared with the fiducial interval, the MIM interval exhibits more accurate coverage with a shorter expected length for all sample sizes. Hence, in terms of coverage probability and expected length, the MIM CI is the best of all.

**Fig 1:**
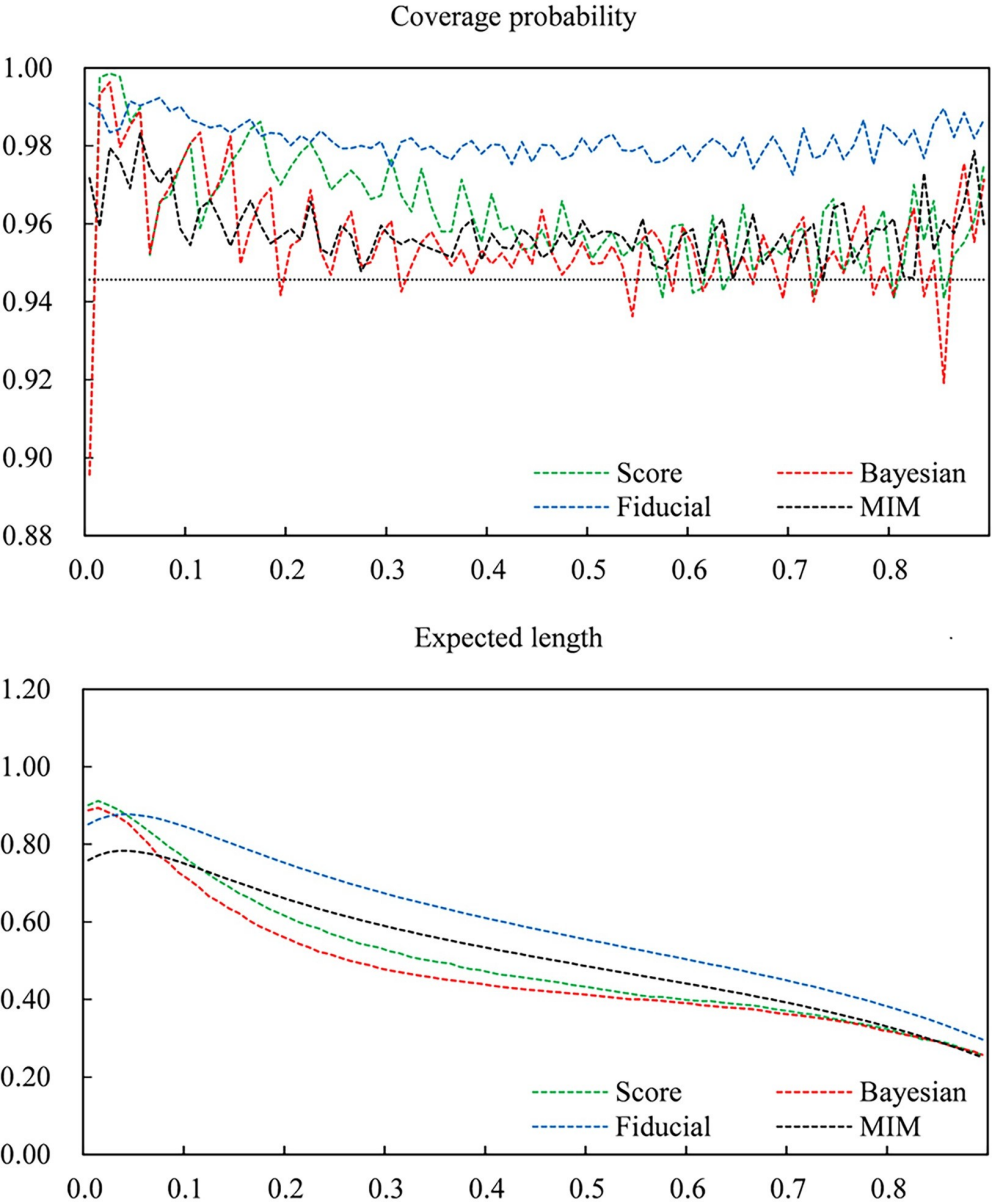
Coverage probabilities and expected lengths of the Score, Bayes, fiducial and MIM confidence intervals versus *P*_12_ for fixed *P*_11_, where *n* = 30 and *P*_11_ = 0.1.

**Fig 2:**
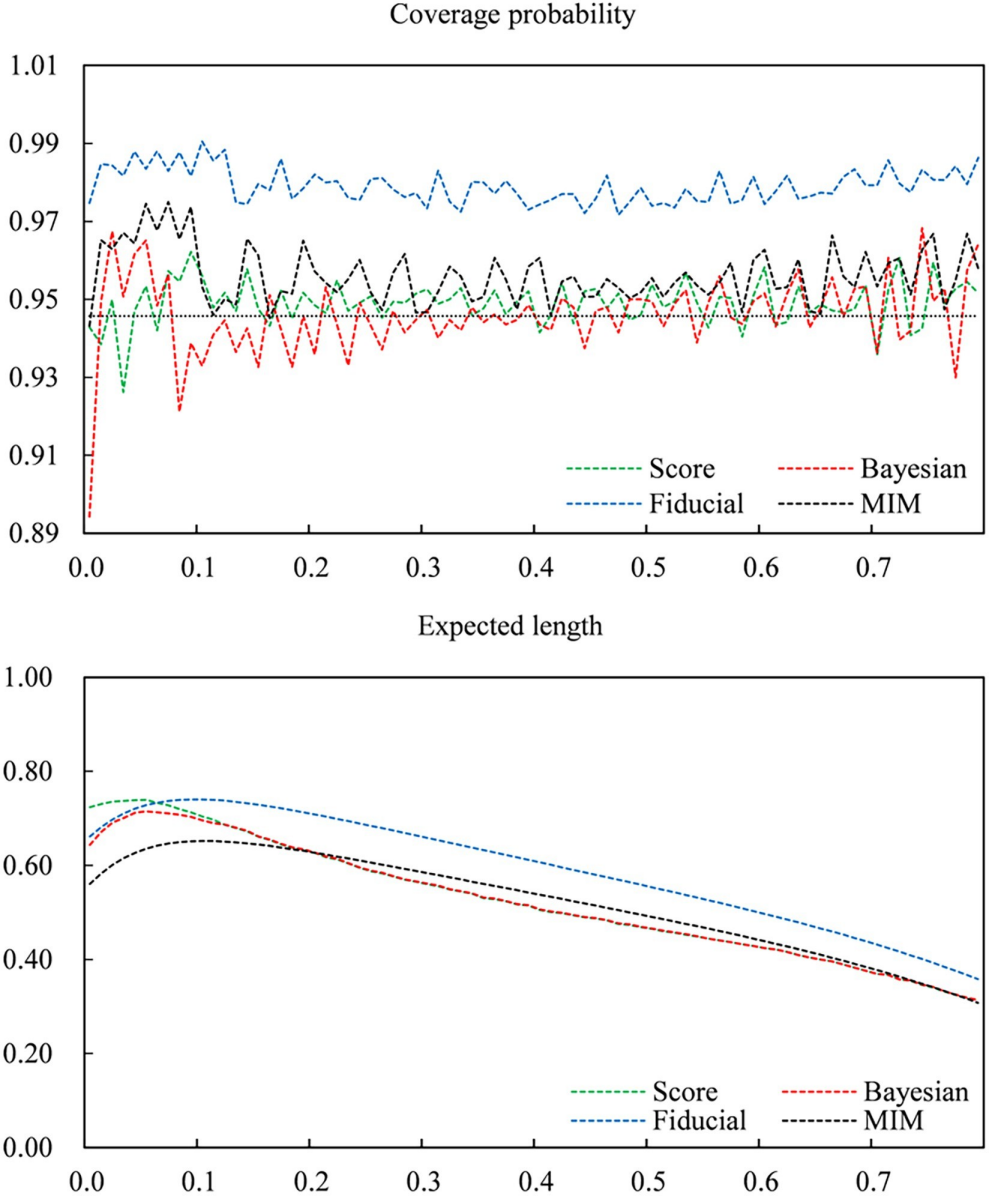
Coverage probabilities and expected lengths of the Score, Bayes, fiducial and MIM confidence intervals versus *P*_12_ for fixed *P*_11_, where *n* = 30 and *P*_11_ = 0.2.

**Fig 3:**
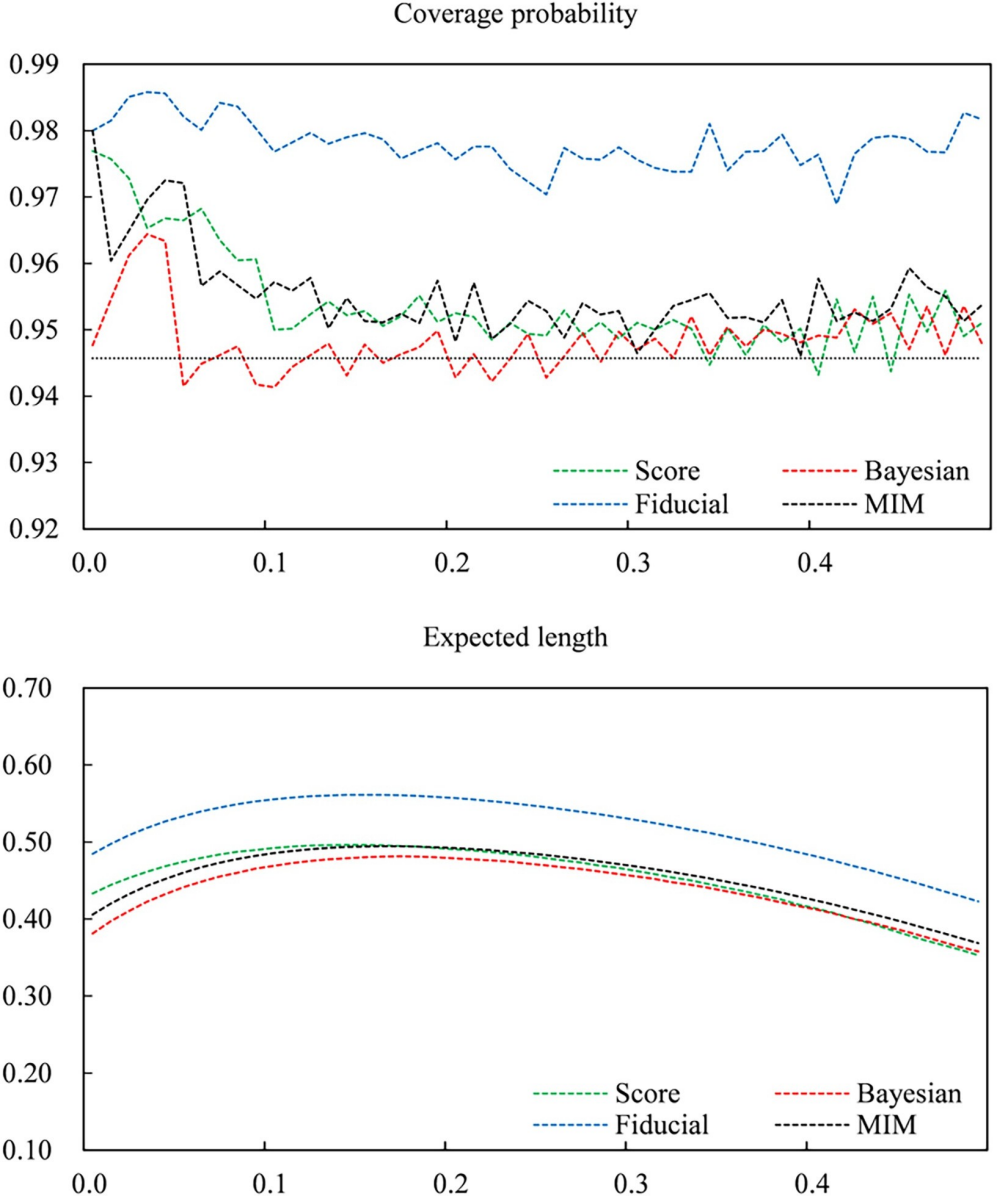
Coverage probabilities and expected lengths of the Score, Bayes, fiducial and MIM confidence intervals versus *P*_12_ for fixed *P*_11_, where *n* = 30 and *P*_11_ = 0.5.

To obtain a better understanding of the different performances of various intervals for small sample sizes. Let *n* = 30. Figs 1–3 give plots of the coverage probabilities and expected lengths of four CIs versus *p*_12_ for fixed *p*_11_ = 0.1, 0.2 and 0.5, respectively. Here, we draw a dashed line (*y =* 0.9457) as the maximum lower bound of the coverage probability. Clearly, the score and Bayesian intervals have coverage probabilities lower than 0.9457 in some cases, especially when *p*_12_ is very close to zero. In contrast, the fiducial approach can always guarantee nominal coverage for all cases, and the MIM CI improves the conservative coverage of the fiducial CI. For expected length, the fiducial and MIM methods have shorter expected lengths than score and Bayesian approaches when *p*_12_ is close to zero. Moreover, although the score and Bayesian CIs have shorter expected lengths in most cases, differences between various expected lengths are small when *p*_12_ becomes larger. Note that the score and Bayesian CIs cannot guarantee the preset coverage probability; hence, the fiducial and MIM intervals are superior to the score and Bayesian intervals.

**Fig 4:**
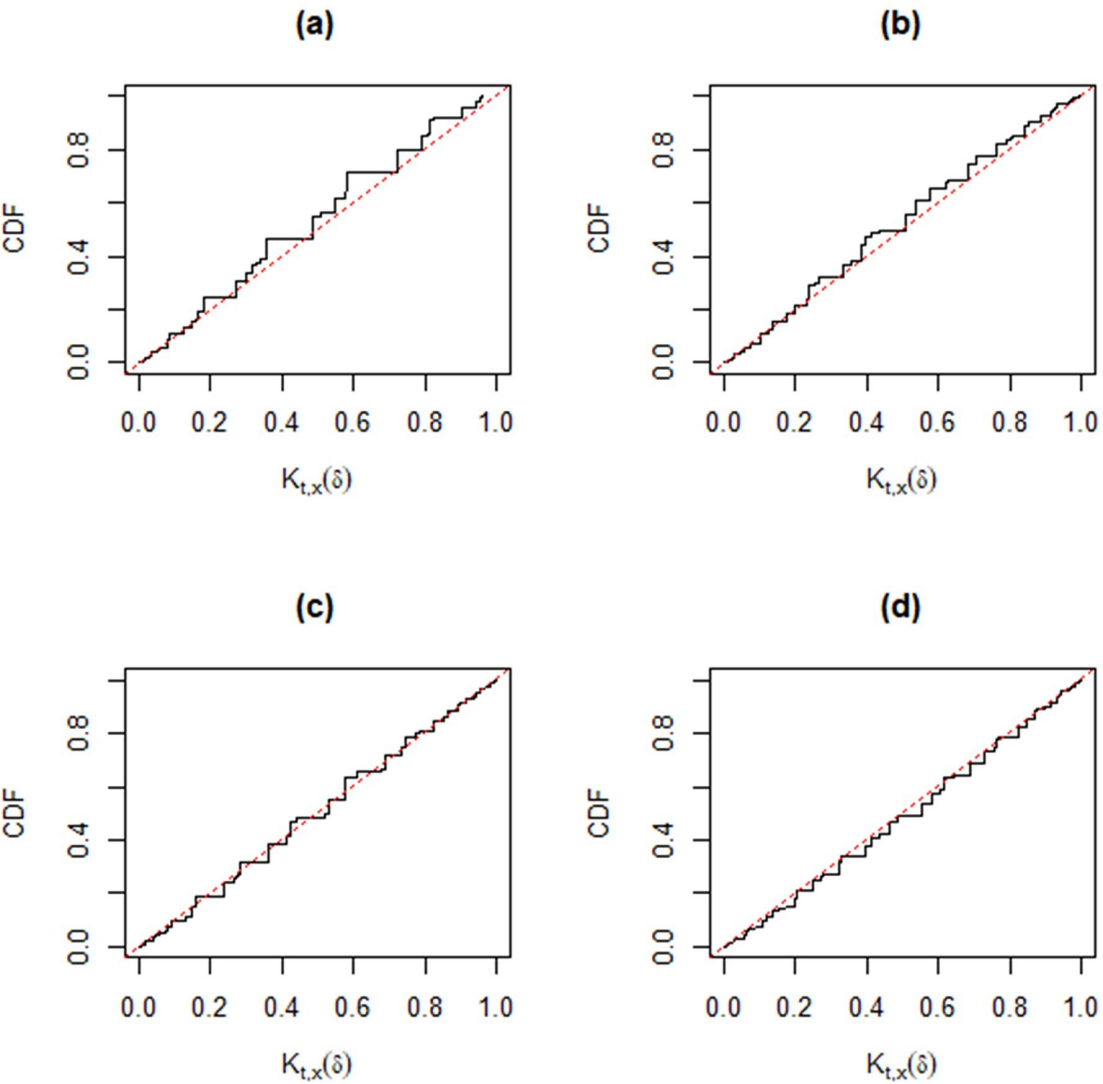
Distribution functions of *K*_*t*,*x*_(*δ*) (black) compared with that of Unif(0, 1) (gray) based on Monte Carlo samples from the trinomial distribution versus *p*_12_ for fixed *n* = 20 and *p*_11_ = 0.1. Panel (a): *p*_12_ = 0.1. Panel (b): *p*_12_ = 0.2. Panel (c): *p*_12_ = 0.3. Panel (d): *p*_12_ = 0.5.

**Fig 5:**
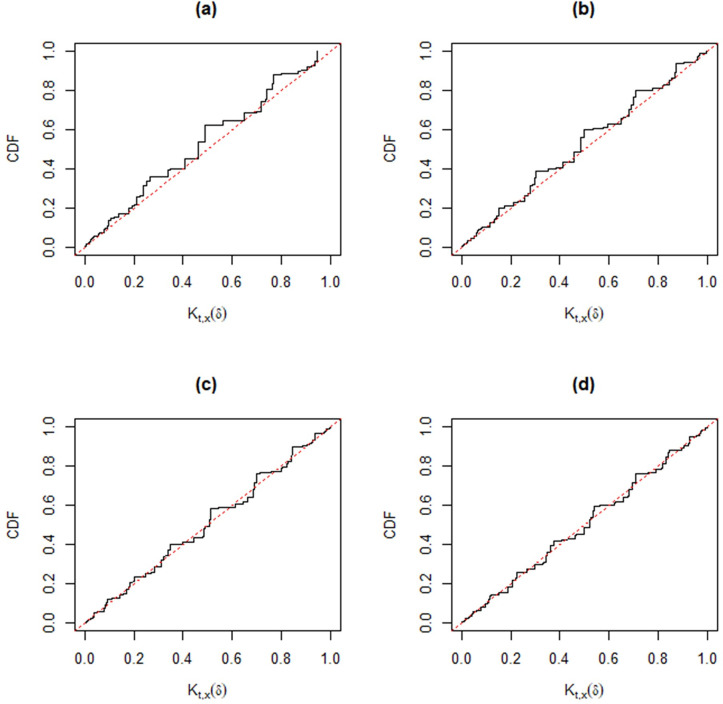
Distribution functions of *K*_*t*,*x*_(*δ*) (black) compared with that of Unif(0, 1) (gray) based on Monte Carlo samples from the trinomial distribution versus *p*_12_ for fixed *n* = 20 and *p*_11_ = 0.3. Panel (a): *p*_12_ = 0.1. Panel (b): *p*_12_ = 0.2. Panel (c): *p*_12_ = 0.3. Panel (d): *p*_12_ = 0.5.

**Fig 6:**
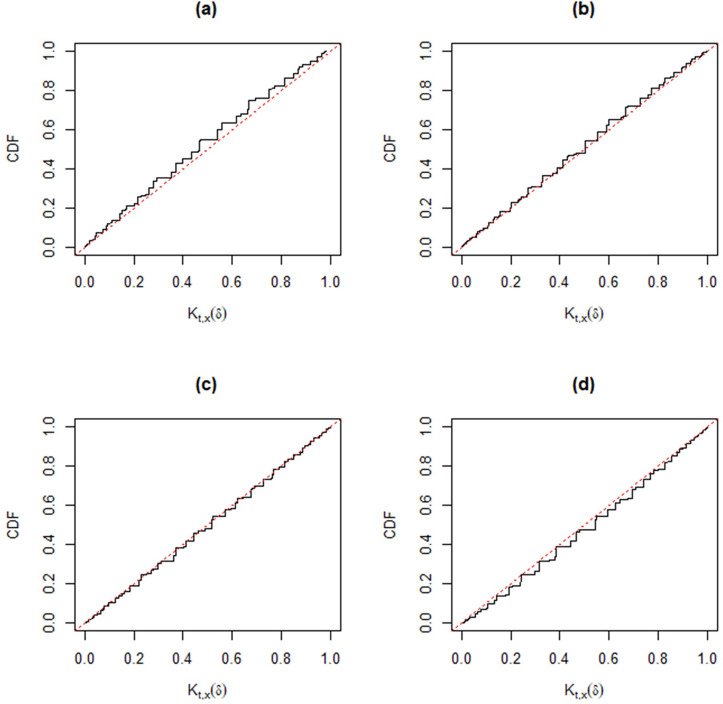
Distribution functions of *K*_*t*,*x*_(*δ*) (black) compared with that of Unif(0, 1) (gray) based on Monte Carlo samples from the trinomial distribution versus *p*_12_ for fixed *n* = 20 and *p*_11_ = 0.5. Panel (a): *p*_12_ = 0.1. Panel (b): *p*_12_ = 0.2. Panel (c): *p*_12_ = 0.3. Panel (d): *p*_12_ = 0.5.

**Fig 7:**
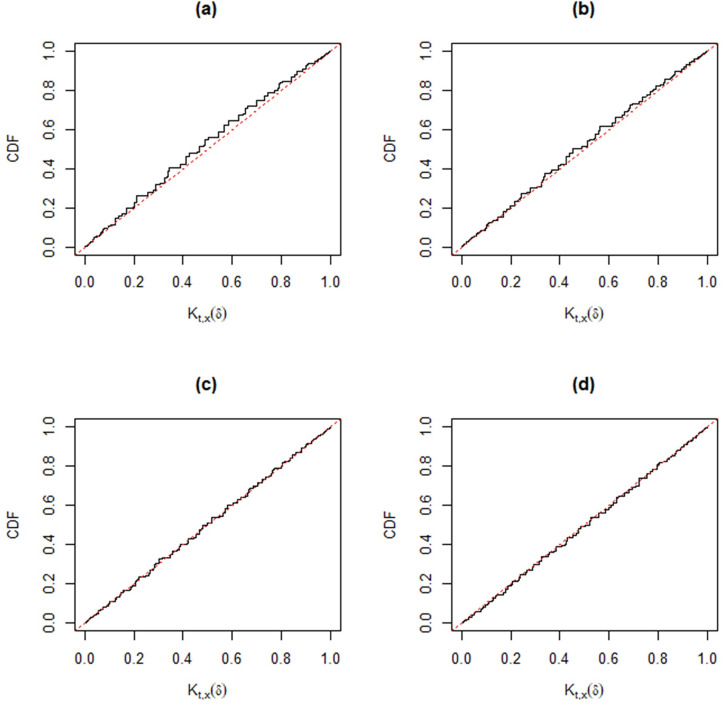
Distribution functions of *K*_*t*,*x*_(*δ*) (black) compared with that of Unif(0, 1) (gray) based on Monte Carlo samples from the trinomial distribution versus *p*_12_ for fixed *n* = 50 and *p*_11_ = 0.1. Panel (a): *p*_12_ = 0.1. Panel (b): *p*_12_ = 0.2. Panel (c): *p*_12_ = 0.3. Panel (d): *p*_12_ = 0.5.

**Fig 8:**
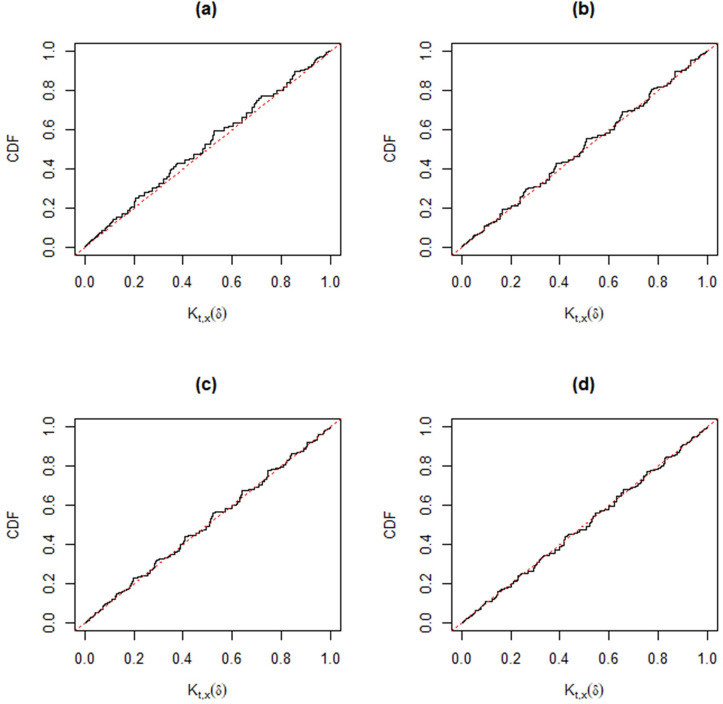
Distribution functions of *K*_*t*,*x*_(*δ*) (black) compared with that of Unif(0, 1) (gray) based on Monte Carlo samples from the trinomial distribution versus *p*_12_ for fixed *n* = 50 and *p*_11_ = 0.3. Panel (a): *p*_12_ = 0.1. Panel (b): *p*_12_ = 0.2. Panel (c): *p*_12_ = 0.3. Panel (d): *p*_12_ = 0.5.

**Fig 9:**
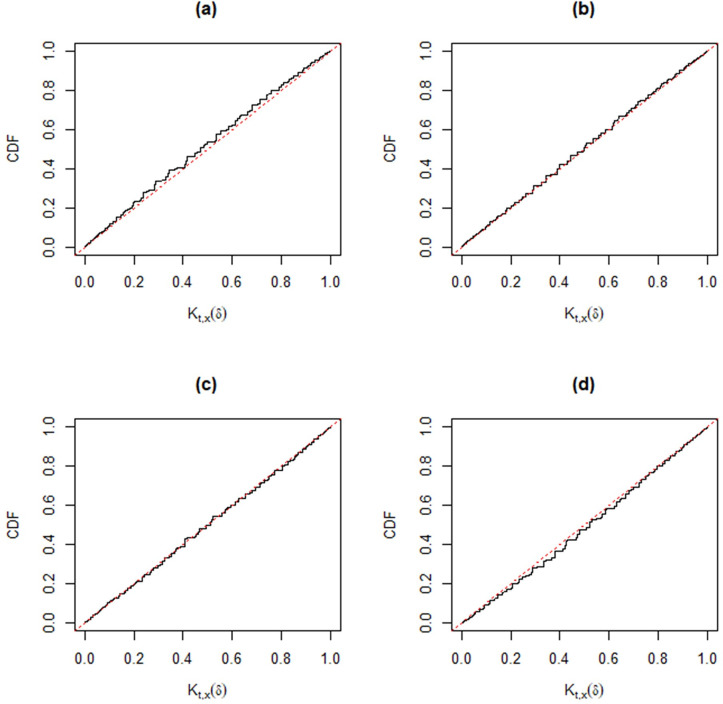
Distribution functions of *K*_*t*,*x*_(*δ*) (black) compared with that of Unif(0, 1) (gray) based on Monte Carlo samples from the trinomial distribution versus *p*_12_ for fixed *n* = 50 and *p*_11_ = 0.5. Panel (a): *p*_12_ = 0.1. Panel (b): *p*_12_ = 0.2. Panel (c): *p*_12_ = 0.3. Panel (d): *p*_12_ = 0.4.

Following [30], when the observed data (*t*,*x*) cannot be separated from the auxiliary variables (*u*’,*v*’), the validity condition *K*_*t*,*x*_(*δ*)~*Unif*(0,1) may not be automatic. However, different from other approaches, we can check the good coverage performance of the MIM method for different parameter settings, *p*_11_ = 0.1 (0.2) 0.5; *p*_12_ = 0.1 (0.1) 0.5; *n* = 20 and 50. In each simulation, we take 10,000 samples. The corresponding Monte Carlo estimators of the distribution function of *K*_*t*,*x*_(*δ*) in Figs 4–9 show that the approximate $pl_{t,x}^{'}(A)$ is valid, that is,

$$\underset{\delta \in A}{\sup}\;P_{t,x|\delta }\{pl_{t,x}^{'}(A)\leq \alpha \}=\underset{\delta \in A}{\sup}\;P_{t,x|\delta }\{K_{t,x}(\delta )\leq \alpha /2\cup K_{t,x}(\delta )\geq 1-\alpha /2\}$$

$$\leq \underset{\delta \in A}{\sup}\;P_{W}\{W\leq \alpha /2\cup W\geq 1-\alpha /2\}=\alpha ,$$,

where *W*~*Unif*(0,1). In particular, from Fig 7–9, the distribution function of *K*_*t*,*x*_(*δ*) is very close to that of Unif(0, 1) for a moderate sample size, hence the MIM method has accurate coverage.

## A real data analysis

We illustrate the application of the proposed methods with a real example. A sample of 156 dairy calves born in Florida, were classified according to whether they had pneumonia within 60 days after birth [4]. Calves that got a pneumonia infection were also classified according to whether they got a secondary infection within two weeks after the first infection cleared up. Table 5 shows the data. Calves that did not get a primary infection could not get a secondary infection, so no observations can fall in the cell for “no” primary infection and “yes” secondary infection. The goal of this study was to test whether the probability of primary infection was the same as the conditional probability of secondary infection, given that the calf got the primary infection.

**Table 5:** Primary and secondary pneumonia infections of calves.

	Secondary infection		
Primary infection	Yes	No	Total
Yes	30	63	93
No	0	63	63
Total	30	126	156

**Fig 10:**
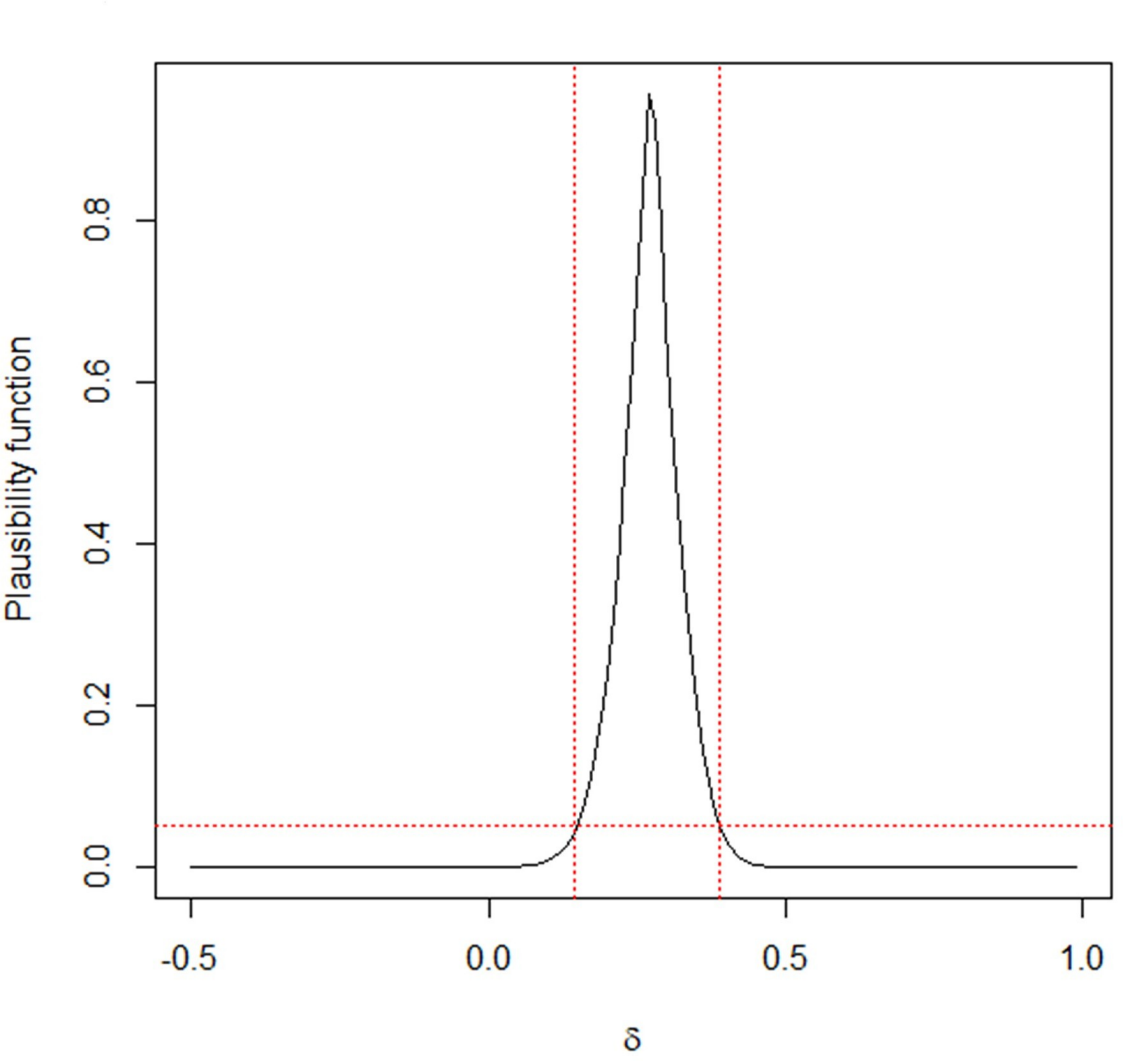
MIM’s Plausibility function of *δ* in the calf immunity data.

Here we used the RD to study the effect of primary infection on the likelihood of developing secondary infection. Under the 95% confidence level, the score, Bayesian, fiducial and MIM CIs for *δ* are (0.15, 0.39), (0.15, 0.39), (0.14, 0.40) and (0.15, 0.39), respectively. Clearly, the lower bounds of the four intervals are all larger than 0. It is suggested that the primary infection of pneumonia should stimulate a natural immunity to reduce the likelihood of secondary infection. Hence, the fiducial and MIM methods work well with calf immunity data. More importantly, the MIM method also provides probabilistic summaries of the information in data concerning the quantity of interest. To be more informative, we plot the plausibility function $pl_{t,x}^{'}(\delta )$, as a function of *δ* in Fig 10. By locating *α* = 0.05 on the vertical axis, we can easily find that the lower bound (0.15) and the upper bound (0.39) are in the MIM CI. Furthermore, the plausibility function shows that each point *δ* in the MIM interval is individually sufficiently plausible. Clearly, no frequentist or Bayesian interval can assign such a meaning to the individual elements it contains. In this sense, the proposed MIM interval is recommended for practical use.

## Discussion

The RD is a comparative measure between the probability of the primary infection and the conditional probability of the secondary infection, given the primary infection. The confidence intervals of the score and Bayesian methods have poor coverage performance for small to moderate sample sizes. In this paper, we propose three valid CIs based on the fiducial, IM and MIM approaches for the RD. The fiducial and IM-based CIs have more accurate coverage performance than the score and Bayesian CIs. Compared with the fiducial approach, IM-based approaches can provide meaningful probabilistic summaries of the information in data concerning the quantity of interest. Moreover, the MIM method uses a randomized IM idea to modify the two inequation associations of IM to an accurate equation model. A real data example shows that the proposed methods work well for the calf immunity data.

Different from other approximate approaches, our MIM solution has the advantage that its approximation precision, which only depends on the simulation times rather than the sample size, may tend to be as high as possible whether the sample size is large or small. Moreover, the IM’s output is posterior-probabilistic in nature and, therefore, has a meaningful interpretation within and not just across experiments. Moreover, our fiducial and IM-based methods are general ideas that can be applied to infer other risk factors, such as the risk ratio. Finally, since the effect of the observed data cannot be separated from the auxiliary variables, there could be interest in the simultaneous prediction of several auxiliary variables. The best choice of predictive random set needs further study.

## Supporting information

S1 Appendix (DOCX)
