# Supplementary material for: Accurate interval estimation for the risk difference in an incomplete correlated 2 × 2 table: Calf immunity analysis
Source: PLoS One. 2022 Jul 22;17(7):e0272007. doi: 10.1371/journal.pone.0272007 (PMC9307212; doi:10.1371/journal.pone.0272007)
Supplement: S1 Appendix — (DOCX) [file pone.0272007.s001.docx]

**Appendix**

**R codes for calculation**

1. fiducial (IM) and MIM intervals for a real data example

f<-function(p,w1,n,n1p,u1) w1*pbeta(p,n1p+1,n-n1p)+(1-w1)*pbeta(p,n1p,n-n1p+1)-u1

g<-function(q,w2,n1p,n11,v1) w2*pbeta(q,n11+1,n1p-n11)+(1-w2)*pbeta(q,n11,n1p-n11+1)-v1

MC<-100000

n<-156

n11<-30

n12<-63

n1p<-n11+n12

u<-runif(MC,0,1)

v<-runif(MC,0,1)

h1<-qbeta(u,n1p,n-n1p+1)-qbeta(v,n11+1,n1p-n11)

h2<-qbeta(u,n1p+1,n-n1p)-qbeta(v,n11,n1p-n11+1)

a<-vector()

for(i in 1:MC){

u1<-runif(1,0,1)

v1<-runif(1,0,1)

w1<-runif(1,0,1)

w2<-runif(1,0,1)

ro1<-uniroot(f,c(0,1),w1=w1,n=n,n1p=n1p,u1=u1)$root

ro2<-uniroot(g,c(0,1),w2=w2,n1p=n1p,n11=n11,v1=v1)$root

delta<-ro1-ro2

a<-c(a,delta)

}

L1<-unname(quantile(h1,0.025))

U1<-unname(quantile(h2,0.975)) #fiducial (IM) interval

L2<-unname(quantile(a,0.025))

U2<-unname(quantile(a,0.975)) #MIM interval

2. Coverage probabilities and expected lengths of fiducial (IM) and MIM intervals

f<-function(p,w1,n,n1p,u1) w1*pbeta(p,n1p+1,n-n1p)+(1-w1)*pbeta(p,n1p,n-n1p+1)-u1

g<-function(q,w2,n1p,n11,v1) w2*pbeta(q,n11+1,n1p-n11)+(1-w2)*pbeta(q,n11,n1p-n11+1)-v1

MC<-10000

n<-30

a1<-vector()

a2<-vector()

a3<-vector()

a4<-vector()

for(n1p in seq(0,n,1)){

for(n11 in seq(0,n1p,1)){

n12<-n1p-n11

n1p<-n11+n12

u<-runif(MC,0,1)

v<-runif(MC,0,1)

h1<-qbeta(u,n1p,n-n1p+1)-qbeta(v,n11+1,n1p-n11)

h2<-qbeta(u,n1p+1,n-n1p)-qbeta(v,n11,n1p-n11+1)

a5<-vector()

for(i in 1:MC){

u1<-u[i]

v1<-v[i]

w1<-runif(1,0,1)

w2<-runif(1,0,1)

ro1<-uniroot(f,c(0,1),w1=w1,n=n,n1p=n1p,u1=u1)$root

ro2<-uniroot(g,c(0,1),w2=w2,n1p=n1p,n11=n11,v1=v1)$root

fai<-ro1-ro2

a5<-c(a5,fai)

}

IM1L<-unname(quantile(h1,0.025))

IM1U<-unname(quantile(h2,0.975))

IM2L<-unname(quantile(a5,0.025))

IM2U<-unname(quantile(a5,0.975))

a1<-c(a1,IM1L)

a2<-c(a2,IM1U)

a3<-c(a3,IM2L)

a4<-c(a4,IM2U)

}

}

data<-data.frame(a1,a2,a3,a4)

N<-10000

p1p<-0.3

phi<-0.1

p11<-p1p-(p1p-phi)*p1p

p12<-p1p-p11

e1<-data[,1]

e2<-data[,2]

e3<-data[,3]

e4<-data[,4]

p1p<-p11+p12

phi<-p1p-p11/p1p

dk1<-0

dk2<-0

for(k in 1:N){

D<-rmultinom(1,n,c(p11,p12,1-p11-p12))

n11<-D[1]

n12<-D[2]

n1p<-n11+n12

j<-n1p*(1+n1p)/2+n11+1

if(e1[j]<=phi&&phi<=e2[j]) {dk1<-dk1+1}

if(e3[j]<=phi&&phi<=e4[j]) {dk2<-dk2+1}

}

a6<-vector()

a7<-vector()

for(n1p in seq(0,n,1)){

for(n11 in seq(0,n1p,1)){

n12<-n1p-n11

n1p<-n11+n12

jj<-n1p*(1+n1p)/2+n11+1

L1<-(e2[jj]-e1[jj])*dmultinom(c(n11,n12,n-n1p),n,c(p11,p12,1-p11-p12))

L2<-(e4[jj]-e3[jj])*dmultinom(c(n11,n12,n-n1p),n,c(p11,p12,1-p11-p12))

a6<-c(a6,L1)

a7<-c(a7,L2)

}

}

cp1<-dk1/N #fiducial (IM) coverage probability

cp2<-dk2/N #MIM coverage probability

el1<-sum(a6) # fiducial (IM) expected length

el2<-sum(a7) #MIM expected length

3. Empirical distribution function of $K_{t,x}(\delta)$

f<-function(p,w1,n,n1p,u1) w1*pbeta(p,n1p+1,n-n1p)+(1-w1)*pbeta(p,n1p,n-n1p+1)-u1

g<-function(q,w2,n1p,n11,v1) w2*pbeta(q,n11+1,n1p-n11)+(1-w2)*pbeta(q,n11,n1p-n11+1)-v1

MC<-10000

n<-100

a1<-vector()

a2<-vector()

a3<-vector()

a4<-vector()

p11<-0.3

p12<-c(0.1,0.2,0.3,0.4)

delta<-(p11+p12)-p11/(p11+p12)

for(n1p in seq(0,n,1)){

for(n11 in seq(0,n1p,1)){

n12<-n1p-n11

n1p<-n11+n12

aa<-vector()

for(i in 1:MC){

u1<-runif(1,0,1)

v1<-runif(1,0,1)

w1<-runif(1,0,1)

w2<-runif(1,0,1)

ro1<-uniroot(f,c(0,1),w1=w1,n=n,n1p=n1p,u1=u1)$root

ro2<-uniroot(g,c(0,1),w2=w2,n1p=n1p,n11=n11,v1=v1)$root

fai<-ro1-ro2

aa<-c(aa,fai)

}

aa1<-mean(aa<=delta[1])

aa2<-mean(aa<=delta[2])

aa3<-mean(aa<=delta[3])

aa4<-mean(aa<=delta[4])

a1<-c(a1,aa1)

a2<-c(a2,aa2)

a3<-c(a3,aa3)

a4<-c(a4,aa4)

}

}

a5<-vector()

a6<-vector()

a7<-vector()

a8<-vector()

for(i in 1:MC){

D1<-rmultinom(1,n,c(p11,p12[1],1-p11-p12[1]))

n1<-D1[1]

n2<-D1[2]

np1<-n1+n2

j1<-np1*(1+np1)/2+n1+1

b1<-a1[j1]

a5<-c(a5,b1)

D2<-rmultinom(1,n,c(p11,p12[2],1-p11-p12[2]))

n3<-D2[1]

n4<-D2[2]

np2<-n3+n4

j2<-np2*(1+np2)/2+n3+1

b2<-a2[j2]

a6<-c(a6,b2)

D3<-rmultinom(1,n,c(p11,p12[3],1-p11-p12[3]))

n5<-D3[1]

n6<-D3[2]

np3<-n5+n6

j3<-np3*(1+np3)/2+n5+1

b3<-a3[j3]

a7<-c(a7,b3)

D4<-rmultinom(1,n,c(p11,p12[4],1-p11-p12[4]))

n7<-D4[1]

n8<-D4[2]

np4<-n7+n8

j4<-np4*(1+np4)/2+n7+1

b4<-a4[j4]

a8<-c(a8,b4)

}

par(mfrow=c(2,2))

plot(sort(a5), (1:MC-1)/MC, type="l", xlab=expression(K[t][","][x](delta)), ylab="CDF",main="(a)", xlim=c(0, 1), ylim=c(0, 1))

abline(a=0, b=1, lty=3, col="red")

plot(sort(a6), (1:MC-1)/MC, type="l", xlab=expression(K[t][","][x](delta)), ylab="CDF", main="(b)", xlim=c(0, 1), ylim=c(0, 1))

abline(a=0, b=1, lty=3, col="red")

plot(sort(a7), (1:MC-1)/MC, type="l", xlab=expression(K[t][","][x](delta)), ylab="CDF", main="(c)", xlim=c(0, 1), ylim=c(0, 1))

abline(a=0, b=1, lty=3, col="red")

plot(sort(a8), (1:MC-1)/MC, type="l", xlab=expression(K[t][","][x](delta)), ylab="CDF", main="(d)", xlim=c(0, 1), ylim=c(0, 1))

abline(a=0, b=1, lty=3, col="red")

4. Plausibility function of the MIM method

f<-function(p,w1,n,n1p,u1) w1*pbeta(p,n1p+1,n-n1p)+(1-w1)*pbeta(p,n1p,n-n1p+1)-u1

g<-function(q,w2,n1p,n11,v1) w2*pbeta(q,n11+1,n1p-n11)+(1-w2)*pbeta(q,n11,n1p-n11+1)-v1

MC<-10000

n<-156

n11<-30

n12<-63

n1p<-n11+n12

u<-runif(MC,0,1)

v<-runif(MC,0,1)

a<-vector()

for(i in 1:MC){

u1<-u[i]

v1<-v[i]

w1<-runif(1,0,1)

w2<-runif(1,0,1)

ro1<-uniroot(f,c(0,1),w1=w1,n=n,n1p=n1p,u1=u1)$root

ro2<-uniroot(g,c(0,1),w2=w2,n1p=n1p,n11=n11,v1=v1)$root

delta<-ro1-ro2

a<-c(a,delta)

}

del<-seq(-0.5, 0.99, 0.01)

gg<-sort(a)

b<-vector()

for(delt in seq(-0.5, 0.99, 0.01)){

p<-sum(gg<=delt)/MC

pp<-1-2*abs(p-0.5)

b<-c(b,pp)

}

plot(del,b,type="l",xlab=expression(delta),ylab="Plausibility function",main="")

abline(h=0.05,lty=3,col="red")

abline(v=0.1460,lty=3,col="red")

abline(v=0.3903,lty=3,col="red")
